# A New Sign Language for Deaf Mutes

**Published:** 1851-10

**Authors:** 


*A New Sign Language for Deaf Mutes.*—The above is the title of a thesis presented to the medical department of the University of Buffalo, in February, 1851, by Albert J. Meyer, and is a valuable and happy suggestion of what must be considered an important improvement in the languages used at the present time by mutes and the blind. The article itself is last in the importance of that which is propounded, and after the proposition of the plan, little is said of striking importance. Upon the whole, it is well written, and expresses the object in a few words, and much to the point.

From the fact that the sense of touch is always existing with life, and more generally perceptible than any other organ of sense, it strongly recommends itself, and doubtless will be adopted as it becomes better known and understood.

